# 

**Published:** 1806-06

**Authors:** 


### LIST OF NEW MEDICAL PUBLICATIONS.

A Practical Treatise on the Diseases of the Stomach and of Digestion; including the History and Treatment of those Affections of the Liver and Digestive Organs, which occur to Persons who return from the East or West Indies, with Observations on various Medicines; and particularly on the improper Use of Emetics. By Arthur Daniel Stone, M.D. 8vo. 6s. boards.

A Compendium of the Anatomy, Physiology, and Pathology of the Horse. By B. W. Burke. 12s. 6d. boards.

### TO CORRESPONDENTS.

Mr. Chartres's Paper has not been received.

We shall be obliged to Mr. Marson for any important medical cases or facts he may meet with in the course of his practice, but we wish to avoid critical controversy.

Mr. Fogo's answer to Dr. Thomas cannot appear in its present form.

In our next Journal, we hope to be able to add the name of an eminent Professor, who has undertaken the Foreign Department.

Communications are received from Dr. Bellamy, Dr. Kinglake, Mr. Simmons, Mr. Clement, Mr. Ring. Mr. Bradley, Dr. Kentish, and the Suffolk Society.

END OF VOL. XV.

### INDEX.

ABDOMINAL viſcera, on diſeaſes of the 481, 573

Abernethy's, Mr. lectures announced 95

Abſtinence, remarkable caſe of 510

Abſorption, on cutaneous 274

Achard's, Mr. experiments on mercury *ib.*

Aculeus's letters to Rowley 286

Adams's, Dr. Report on inoculation, &c. 195

———, treatiſe on morbid poiſons, announced 296

——— obſervations on quackery 334

——— caſe of yaws 382

——— letter to, on cancer 393

Agaricus campeſtris, on the 247

Agrimony, botanical deſcription of 269

Agues, remedy for 265

———, on ammonia preparata in 333

Air, on purifying the 375

———, foul in oil ciſterns, nature of 391

Alder, Mr. on yellow fever 40

Alexander's, Mr. caſes of chorea ſancti viti 125

Alkalis, ſolution of metallic oxyds in 295

Allen's, Mr. lectures, announced 199

Amelia, yellow fever on board the ſhip 17

Amenorrhœa, on 141

America, on vaccination in 313

American elephant, deſcription of the 486

Ammonia preparata, its efficacy in intermittents 333

Anderſon, Mr. on vaccination 137

Anderſon's, Dr. account of vaccination in India 525

Anecdotes 366, 370

Aneuriſm, on 476

Angina pectoris, caſe of 380

Animal fluids, analyſis of 280

——— deſcription of a foſſil 391

Anti-vacciniſts, replies to, and obſervations on 159, 231, 283, 445, 525

Apoplexia hydrocephalica, caſe of 322

Arbutus, botanical deſcription of the 71, 261

Aretæus on the morbus cardiacus 378

Armiger's, Mr. lectures announced 94

Armſtrong, Dr character of 19

———, Mr. on inoculation 233

Army, on the medical eſtabliſhment of the 88

Arnold's, Dr. treatiſe on inſanity, announced 296

Arſenical ſolution in lepra, on 297

———, its efficacy in periodic head-ach 506

Arteries, experiments on the 463

Aſarabacca, botanical deſcription of 268

Babington's, Dr. lectures announced 199

Badham's, Dr. lectures announced 95

Baillie, Dr on cancerous hydatids 395

Ballhorn, Dr. on the tubercles of conſumptive perſons 30

Ballin's, M. caſe of abſtinence 510

Balls, new inſtruments for extracting 455

Barbadoes, ſingular diſeaſe at 561

Bark, uſes of the oak 69

——— in ſcarlatina anginoſa, on 493

Bartlett, Mr. on monſtroſity 249

Baſilic powder, on the 432

Bath waters, their uſe in iſchias 565

Beatty, Mr. on the Death of Lord Nelſon 72

Beddoes, Dr. on conſumption announced 584

Bees, advice reſpecting 67

Bilious indigeſtion, account of a work on 214

Binns, Dr. on the ſcarlatina anginoſa 489

Birch, Mr. obſervations on the queries of 304

Biſhop, Mr. on the management of leeches 198

Biſmuth, medical uſe of the white oxyd of 563

Back's, Dr. caſe of angina pectoris 380

Blackbind weed, botanical deſcription of 68

Bladder, caſe of laceration in the 153

Blair, Mr. on the cow-pox, announced 392

——— on Dr. Rowley's ox-faced boy 445

——— on the vaccine conteſt 529

Blizard's, Sir W. lectures announced 94

Bone-ſetters, ſhocking practices of 553

BOOKS, CRITICAL ANALYSIS OF NEW MEDICAL.—Enquiry into the Origin, Symptoms, and Cure of Conſtitutional Diſeaſes, by Wm. Lambe, M. D. 74 Commentaries on the Treatment of Scirrhi and Cancers, by Wm. Thomas 79. Inoculation for the Small-pox vindicated, by Geo. Lipſcombe, 81. Obſervations on the Nature and Cure of Fevers and Diſeaſes of the Weſt and Eaſt Indies, by T. Clark, 83. Obſervations on the Nature and Cure of Gout, by James Parkinſon, 175. Obſervations on the Utility and Adminiſtration of Purgative Medicines, by James Hamilton, M. D. 182. A Practical Account of a Remittent Fever, by T. Sutton, M. D. 189. Anfwer to Dr. Mofeley, containing a Defence of Vaccination, by John Ring, 191. The Edinburgh Medical Journal, No. 5, 171. Reply to the Anti-vaccinifts, by James Moore, 283. Letters to Dr. Rowley, by Aculeus, 286. Obfervations on Vaccine Inoculation, by Henry Frafer, M. D. 288. Remarks on the Report of M. Chaptal, &c. by J. C. Smith, M. D. *ib.* Effay on the Difeafes incident to Lafcars by W. Hunter, A. M. 289. Vaccinæ Vindicæ, by R. J. Thornton, M. D. 290. The Hiftory and Treatment of the Difeafes of the Teeth, by J. Fox, 371. Reply to Dr. J. C. Smith, by John Johnftone, M. D. 373. Memoirs of the Medical Society of London, vol. vi. 377, 563. Treatife on the Origin, Progrefs, Prevention, and Treatment of Confumption, by J. Reid, M. D. 460. Treatife on the Procefs employed by Nature in fuppreffing the Hæmorrhage from divided Arteries by J. F. D. Jones, M. D. 463. Effay on the Effects of Carbonate of Iron upon Cancer, by R. Carmichael, 477. A Practical Treatife on various Difeafes of the Abdominal Vifcera, by C. R. Pemberton, M. D. 481, 573. Notes on the Weft Indies, &c. by G. Pinckard, M. D. 561. A Practical Treatife on the Difeafes of the Stomach, by A. D. Stone, M. D. 576.

BOOKS, NEW MEDICAL ANNOUNCED. Dr. Reid on Confumption, 96. Mr. Fox's 2d volume upon the Teeth, *ib.* Dr. Rufh's Works, *ib.* Dr. Hutchinfon on Ulcers, *ib.* Mr. Saunders's Anatomy of the Human Ear, 199. Dr. Harrifon on the State of the Practice of Phyfic, *ib.* Mr. Hunt's Anatomical Speculations, *ib.* Dr. Henderfon's Tranflation of M. Cabanis fur la Reforme de la Medicine, 200. Dr. Trotter on Bilious Indigeftion, 214. Dr. Arnold on Infanity, 296. Dr. Adams on Morbid Poifons, *ib.* Dr. Pinckard's Account of the Expedition to the Weft Indies, *ib.* Dr. Hamilton on Gout, 391. Mr. Creafer on the Medical Application of Galvanifm, 392. Dr. Clarke on Pregnancy, 488. Dr. Willan on the Cow-pox, *ib.* Dr. Clarke on the Difeafes of the Army, *ib.* Mr. Hutchinfon on the Tartrite of Antimony, *ib.* Mr. Royfton on the Medical Literature of England, 583. Dr. Beddoes on Confumption, 584.

Boftock Dr. on a ftearoid tumour 276

——— on animal fluids 280

——— cafes of diabetes 569

Botanical defcription of Britifh plants 66, 261

Bottle, how to prepare a luminous 94

Bowels, cafe of conftipation of the 325

Bowels, cafes of inflammation of the 542

Boy, cafe of one who became blue 381

——— obfervations on the pretended ox-faced 64, 445

Brain, on dropfy of the 122

Brande's Mr. experiments on guaiacum 294

Brookes's Mr. lectures announced 95, 487

Brown Dr. on the fyftem of 91

———, anfwer to 40

Brown, Mr. on yellow fever 560

Bruifewort, botanical defcription of 263

Buck wheat, botanical defcription of 67

Bug-bites, errors concerning 520

Burns Mr. on the human ovum 242

Burns, on the ufe of ol. tereb in 146

———, on the heating treatment of 342

———, on cold water in 458

Cachexia Africana, on the 142

Calculous complaints, remedy for 262

Calomel, its ufe in yellow fever 19

Caldwell's Dr. cafe of offified fœtus 279

Cambridge, on medical degrees at 295

Canada, ftate of vaccination in 391

———, difeafes in 446

Cancer, on the treatment of 79

———, remedy for 265

———, on the hydatid exiftence of 393

———, effects of carbonate of iron on 477

———, letter to Mr. Home on 500

Cantharides in gleet and leucorrhœa, on 415

Carlifle, Mr. on the mufcles of fifhes 95

———, lectures announced *ib.*

Carmichael on carbonate of iron in cancer 477

Carpue's Mr. lectures announced 487

Cayley Dr. on opium in coftivenefs 336

Celfus on the morbus cardiacus 379

Chandler's Mr. cafe of laceration of the bladder 153

———, cafe of hernia 155

Chevalier's Mr. account of a new varnifh 391

———, lectures announced 95

Chickweed, botanical defcription of 264

Chlorofis, on 186

Chorea fancti viti, on the 119

———, on purgatives in 185

Clark, Mr on the nature and cure of fevers 83

Clarke's Dr. lectures announced 95

———, Dr. E. on difeafes of the army announced 488

———, on vaccination 255, 426

Cline's Mr. lectures announced 109

———, obfervation on cancerous hydatids 399

Coal mine, fingular difeafe among labourers in a 93

Conftitutional difeafes, on 74

Cold, on catching 91
—— bathing in yellow fever, on 17
Coleman's Mr. lectures announced 199
Conftantinople, account of the plague at 113
Confumption, on the prevention and treatment of 460
——, on arfenic in 299
Confumptive perfons, on the fœtid tubercles of 30
Contraction of the lower limbs, on 326
Convulfions, on cold water in 52
Convulfive affection, curious cafe of 126
Cooke's Dr. lectures announced 94
Cooper's Mr. lectures announced 199
Copland, Mr. on the virtues of muriatic acid 570
Copper pricipitated by tin 582
Cork, population and hofpitals at 357
Cornfpurry, botanical defcription of 267
Correfpondents to, 96, 200, 296, 392, 483, 584
Coftivenefs, fingular cafe of 100
——, on opium in 336
Couching needle, on the 103
Cow inoculated with variolous matter 48
Cowpox, addrefs to the inhabitants of Yarmouth, on 92
——, on the origin of the 195, 382
——, inftitution at Nottingham proceedings of the 251, 426
——, on the merits of 301
——, on fpurious 308
Cow-pock matter, doubtful origin of 539
Cow-pox appears at a farm 582
—— fketch of a new theory of 571
Coxe, Dr. reply to 119
Craniology, account of Dr. Gall's fyftem of 201
Creafer, Mr. on vaccination 306
Croonian lecture, account of the 95
Crotchet, cafe requiring the ufe of the 1
Croup, on chronic 507
Crufta lactea, on 33
Cuming's Dr. cafe of tinea capitis 43
—— trifmus 44
—— anfwer to Mr. Thomas 224
—— on the ufe of nitre in fphacelus 504
Curry's Dr. lectures announced 199
Cutaneous abforption, on 274
—— diforders, remedy for 270
——, treatment of 300

Dafhwood's Mr. cafe of tetanus 496

Davies's Mr. cafes of phthifis 346

Dawfon's Mr. cafe of incipient procidentia uteri 21

De Carro's, Dr. zeal for vaccination 390

—— hiftory of vaccination 516

Dennifon's Dr. lectures announced 94

Desfontaine's Dr. cafe of difeafed liver 26

Defgennettes Dr. on the plague 42

Diabetes mellitus, cafe of 276

—— cafes of 569

Digitalis in confumption, efficacy of 32

Difeafe caufed by an infect in the liver 26

——, account of an extraordinary 93

Difeafes, on conftitutional 74

——, on converfion of 131

—— in London, fee London

—— in the Liverpool difpenfary 388

—— of the abdominal vifcera 481, 573

——, on vaccine and variolous 219

——, in an eaftern diftrict of London 580

Dog-diftemper, account of 582

Dog's mercury, botanical defcription of 71

Dolor faciei, cafe of 233

Dropfy, remedy for the 265

—— cafe of 501

—— of the brain, on 122

Dublin, ftate of vaccination in 319

—— infirmaries at 357

Dunning's Mr. cafes of fpina bifida 236

—— on rupturing the vaccine veficle 238

Dyfentery, on 85

Dyfon's Mr. cafe of inverted uterus 355

Dyfpepfia, cafes of 194

—— on exercife in the cure of 273

—— remedy for 564

Earneft's Mr. cafe of acute rheumatifm 45
—— cafe of coftivenefs 100
—— on typhus fever 239
Edinburgh Medical Journal reviewed 271
Edwards's, Dr. lectures announced 95
Egypt, on the plague in 114
Elephant, account of the American 486
Elephantiafis of Barbadoes, defcription of 561
Empiricifm, origin and progrefs of 362
Enthyema mercuriale, effay on 280
Epilepfy, empirical remedy for 355
Eryfipelas, fimilarity between fcarlatina and anginofa and 492
Eryfipelatous eruptions, remedy for 264
Exercife in the cure of dyfpepfia, on 273
Experiments on foul air in oil cifterns 391
—— on cutaneous abforption 274
—— on animal fluids 280
—— Galvanic 294
—— on James's powder 294
—— on guaiacum ib.
—— on the torpedo 295
—— on metallic oxyds ib.
—— on divided arteries 463
Eyes, on inflammation of the 194

Falconer, Dr. on the morbus cardiacus 377
——, Dr. on the Bath waters 565
Fafhion, on 91
Faulkner, Dr. on dyfpepfia 273
Febris intermittens, on the cure of 333
Fever, ufe of fpirit of turpentine in yellow
——, on the treatment of yellow 17
——, on the natture and cure of 83
——, on purgative medicines in typhus 183

Fever, account of a remittent 189
——, cafe of typhus 239
——, recantation of Dr. Rufh on 271
——, on the cure of intermittent 333
—— account of a malignant one at Quebec 446
—— on bark in 494
Field's, Mr. cafe of inteftinal ulceration 386
Fifhes, on the mufcles of 95
Fiftulous finufes, remedy for 324
Fits, on oxygen gas in 582
Fluids, analyfis of animal 280
Fœtid grains in the fputa of confumptive perfons, on 33
Fœtus, on the nourifhment of the 229
——, found in the abdomen of an aged woman 276
——, cafe of an offified 279
——, cafe of extra-uterine 385
Fogo, Mr. reply to 141
Forbes, Dr. on empiricifm 362
Foffile animal, account of a 391
Fothergill's Dr. cafe of extra-uterine fœtus 385
——, account of difeafes 386, 488 578
Fowler's Dr. Mineral folution, on the effects of 297
Fox's Mr. treatife on the teeth announced 96; reviewed 371
——'s lectures announced 199
Fractures of the thigh, on 8, 339
Framboefia guinianfis, cafe of 382
Frampton's Mr. lectures announced 94
France, ftate of vaccination in 315
——, cow-pox introduced into the Ifle of 523
Frafer, Dr on his notion of the cow-pox 195
——, his cafe of diabetes mellitus 276
——, obfervations on vaccine inoculation 288
Friction, conftipation of the bowels removed by 325

Gall's Dr. fyftem of craniology, account of 201
Galls on oak leaves, their ufe 69
Galvanic experiments 294
—— action how augmented 487
Galvanifm, cafes of the effects of 150
——, applied to diftinguifh real from apparent death 291
Gapper's Mr. cafe of hydrocephalus internus 380
Gaftrodynia, on a cafe of 216
Girdleftone's Dr. addrefs on the cow-pox 92
——, on the mineral folution in lepra 397
Gland, affection of the proftate 487
Gleet, ufe of cantharides in 415
Good Hope, vaccination eftablifhed at the Cape of 524
Goodwin's Mr. cafe of ftone and gravel 35
Gout, from morbific matter 78
Gout, on the nature and cure of 175
Gravel, cafe of 35
Greeks, of the plague among the 113
Greenftone, pretended virtues of a 336
Griffith's Mr. account of a wormfhell 391
Grivel's M. account of a remarkable fœtus 276
Groin, ulcerated tumour in the 221
Guaiacum, experiments on 294
——, its ufe in gout 181
Gunning's Mr. lectures announced 392, 584
Gunfhot wounds, ufe of nitre in 97
——, inftrument for extracting balls from 455
Gutta ferena, cured by galvanifm 151
Guy's hofpital, lectures at 198

Halefworth, account of the difpenfary at 549
Hæmatemefis, on the utility of purgative medicines in 186
——, cafe of 387
Hæmorrhoidal affections, remedy for 70
Haighton's Dr. lectures announced 199
Hall Mr. on the fcrew tourniquet of 7, 112, 149
—— Dr. on Hydrophobia 409
——, on a cafe of abftinence 510
Hamilton's Dr. lectures announced 94
—— on purgative medicines 182
—— on gout announced 391
—— account of the difpenfary at Halefworth 549
Hardman, Mr. on the fponge peffary 235
Harrifon Dr. on the practice of phyfic announced 199
Harrifon's, Dr. correfpondence, remarks on 557
Harrold Mr. on fractures of the thigh 8, 149
——, on crufta lactea 34
——, on ol. tereb. in burns 146
——, on the fcrew tourniquet 149
——, his machine for fractured thighs 439
——, on cold water in burns 358
Hatchett, Mr. on artificial tanning 295
Hawker's, Mr. account of a foffil animal 391
Head-ach, cafe of periodic 506
Headington's, Mr. lectures announced 94
Heart, peculiar morbid appearance of the 386
Heberden, Dr. on bark in fevers 494
Hemorrhage from divided arteries, on 463
——, remedy for 270
Henderfon's, Dr. Tranflation of Cabanis fur la Reforme de la Medicine announced 200
Hepatic difeafe, cafe of obftinate 381
Herman, Mr. on Galvanic conductors 487

Hernia, case of 155

———, case of scrotal 381

Hill, Mr. on the contraction of the lower limbs 326

Hip-cases in the Bath hospital 566

Hippocrates on the medical profession 364

Hoffman, on costiveness 102, *note*

Holst, Dr. on yellow fever 16

Home, Mr. letter to 500

Horne's, Mr. case of particular affection of the prostate gland 487

Hooper's Dr. lectures announced 487

Horse, cow-pox procured from the 195, 382, 517

———, cow-pock traced from a 582

Hospitals in Ireland, observations on the 242, 353

Huggan, Mr. on cold water in convulsions 52

———, Dr. his cases of secondary small-pox 434

Humboldt, M. on the torpedo 295

Hunt's, Mr. anatomical speculations announced 199

Hunter, Mr. on the diseases of Indian Seamen 289

Hutchinson, Dr. on ulcers announced 96

———, Mr. on the tartrite of antimony, announced 488

Hydatids in cancer, observations on 393

Hydrocephalus internus, cases of 28, 380

———, symptoms of 388

Hydrophobia, on 409

Hyslop, Mr. receives the Jacksonian Prize 487

Hysteria, on purgative medicines in 187

Icterus calculosus, virtue of muriatic acid in 570

Idiosyncrasy, case of 444

Incipient procidentia uteri, case of 21

India, state of vaccination in 518

India, on vaccination in 525

Indians, vaccination among the North American 313

Indies, on the diseases of the West and East 83

Indigestion, account of an intended work on 213

Inoculation, Mr. Ring on 56, 313, 516

———, report of the medical council on vaccine 107

———, Dr. Wood on 135

——— hospitals, statement of the 195

———, cases of 233

———, Nottingham institution for 251

———, comparative merits of cow-pock and small pox 301

———, on vaccine 350

——— at Liverpool 536

Insanity, remarkable case of 510

Insect in the liver, case of 26

Instrument for extracting balls, description of 455

Intestinal ulceration, case of 386

Intestines, on the peristaltic motion of the 102

Ipecacuanha in consumption, on 32

———, substitute for 268

Ireland, on the hospitals of 242, 353

Iris, on the division of the 4

Iron, effects of the carbonate of 477

Ischias, use of bath waters in 565

Italy, success of vaccination in 524

Itch, remedy for the 263

Jacksonian prize 1806 583

Jacobi's, Dr. cases of tussis convulsiva 222

Jamaica, on the climate and diseases of 41

James's, Dr. powder analysed 294

Jardine, Mr. on Dr. Rush's recantation 270

Jaundice, use of bruise wort in the 263

———, remedy for 270

Jenner, Dr. on the treatment of 284

Jennerian society, report of the royal 107

——— in Somerset, report of the 309

Johnstone's, Dr. reply to Dr. Smyth, reviewed 373

Joints, on gouty affections of the 178

Jones, Dr. on hæmorrhage, reviewed 463

Kentish Dr. on the treatment of burns 348

Kidneys, on diseases of the 573

Kilkenny, population of 357

Kinglake Dr. on variolous and vaccine disease 48

———, remarks on his theory of the gout 181

———, answer to 219

———, on burns and scalds 342

———, on the origin of the cow-pock 539

Klaproth Mr. on the solution of metallic oxyds 295

Knawell, botanical description of 263

Knot grass, botanical description of 67, 262

Kopp's Dr. case of dolor fæciei 230

Laceration of the bladder, case of 153

Lambe, Dr. on constitutional diseases 74

Lambert Mr. D. account of 583

Langford Mr. extraordinary case of 439

Lascars, on the diseases of 289

Lawrence's Mr. lectures announced 95

LECTURES announced: At the London Hospital, 94. At St. Bartholomew's Hospital, *ib.* The Croonian, by Mr. Carlisle, 95. Dr. Badham's, on Medicine and Chemistry, *ib.* Mr. Brooke's, on Anatomy, Physiology, and Surgery, 45, 487. Dr. Clarke's, on Midwifery, *ib.* Mr. Chevalier's, on Surgery, *ib.* Mr. Macartney's, on Comparative Anatomy, *ib.* Mr. Pearson's, on Surgery, 96. Dr. Reid's, on the Theory and Practice of Medicine, 96, 48

Mr. Taunton's, on Anatomy, Phyfiology, and Surgery 96, 487. At St. Thomas's and Guy's Hofpitals, 198. Mr. Milburne's, on Anatomy, Phyfiology, and Surgery, 199. Mr. Moor's, on the Teeth, *ib.* Mr. Gunning's, on Surgery, 392. Mr. Thelwall's, on Elocution, *ib.* Mr. Carpue's, on Anatomy and Surgery, 487. Dr. Hooper's, on the Theory and Practice of Phyfic, Materia Medica, and Pharmaceutical Chemiftry, *ib.*
Lee, Mr. on fcrotal hernia 381
Leeches, method of treating 198
Leg, cafe of ulcerated 217
Lemons, falt of, how made 267
Lepra, effects of mineral folution in 297
Leprofy of the Jews, on the 385
Leroy, Dr. on the epilepfy 335
Lettfom's Dr. cafe of hepatic difeafe 381
Leucorrhœa, on cantharides in 420
Limbs, on contraction of the lower 326
Limerick, population of 357
Lime water in confumption, on 32
Lipfcombe's Mr. vindication of the fmall-pox inoculation, reviewed 81
Lithiafis, power of muriatic acid in 570
Lithotomy on the pofition of patients in 569
Liver, cafe of difeafed 26
——, on difeafes of the 87
——, remedy for obftruction of the 270
Liverpool dyfpenfary, ftatement of difeafes in the 388
——————, vaccine inoculation at 536
Lobftein, M. on the nourifhment of the fœtus 229
London hofpital, lectures at 94
————, monthly report of difeafes in 90, 193, 291, 386, 483, 578
————, difeafes and cafualties of, for 1805 200
————, memoirs of the medical fociety of 377, 563
Luminous bottle, account of a 94
Luxation of the olecranon, on a 3

Macartney's lectures announced 95
Mac Mullin Dr. on erythema mercuriale 280
Madifon's Bifhop account of the mammoth 486
Mammoth, defcription of the *ib.*
Marafmus accompanies hydrocephalus 185
Marcet's Dr. lectures announced 199
———— on the white oxyd of bifmuth 563
Marfhall Dr. on the origin of the cow-pox announced 382
Medical literature, intended work on 583
———— profeffion, intended reform in the 93
————, Lectures, fee LECTURES
————, publications monthly, lift of 392, 488, 584
Medical, fee BOOKS
———— and phyfical intelligence 92, 195, 294, 390, 486, 582
———— fociety of London, review of the memoirs of the 377, 563
————, reform in 258
Medicines, on purgative 182
————, patent ones, a difgrace 369
Menftrual flux, on the 187
Mercurialis, botanical defcription of 71
Mercury, experiments on 274
————, its effects on the teeth 372
————, on the beft mode of introducing 576
Merriman's Mr. anfwer to Rowley 231
Metal, defcription of a new 390
Metallic tractors, quackery of 369
Milburne's Mr. lectures announced 199
Mineral acid gas, on the difcovery of the powers of 374
———— folution, its effects in lepra 297
Monftrosity, on 250
Moore's Mr. reply to the antivaccinifts 283
————, lectures announced 199
Morbus cardiacus, on the 377
———— facer, cafe of 126
———— mortality, bill of 200
Mofeley, Dr. anfwer to 56, 165, 191
Mucus, on 281
Muriatic acid, conftituent principles of 94
——————, lithontriptic power of 570
Mufhrooms, fatal effects of eating 247

Namlat Mr. on vaccine and variolous difeafes 219
Nævæ Maternæ, on the 249
Needle, on the couching 103
Negro cachexy, on 141
Nelfon (lord), account of the death of 72
Nephritis, on opium in 503
Nervous diforders, on 195
Newcaftle, progrefs of vaccination at 135
Nicolanum, a new metal, account of 390
Night, method of obtaining light in the 94
*Nine*, on the French word 277
Nitre in gun-fhot wounds, on 97
—— fphacelus, on 504
Noble, Mr. on the yellow fever 17
Noli me tangere, cured by carbonate of iron 478
Nottingham, proceedings of the cow-pox inftitution at 251

Oak, defcription and virtues of the 279

Oil cifterns, on the foul air in 391

Ol. Terebinth. in burns, on 146

Olecranon, on a luxation of the 3

Opiates, their efficacy in rheumatifm 47 *note*

Opifthothotonos, cafe of 382

Opium, its effects in coftivenefs 336

————, in nephritis 502

Opium, its effects in inflammation of the bowels 544
Ophthalmia, prevalence of 581
Orpine ftonecrop, botanical defcription of 265
Offified fœtus, cafe of 279
Ovum, on the formation of the human 271
Ox-faced boy, on the pretended 64
Oxyds, on the folution of metallic 295
Oxygen gas, its efficacy in fits 582
Oxygenous gas produces falivation 274
Ozanne Chriftopher, a quack, account of 366

Pacchiani M. on muriatic acid 294
Palfy, on the 47
Patent medicines, danger of 369
Paralyfis, cafe of rheumatism ending in 45
Paris, botanical defcription of the herb 68
Parkinfon Mr. on the nature and cure of the gout 175
Parry, Dr. on a cafe of vaccination 521
Patterfon, Dr. on chorea fancti viti 119
Pearfon's, Mr. lectures announced 96
Pearfon, Dr. on a remarkable fœtus 277
Pemberton, Dr. on difeafes of the abdominal vifcera 481, 573
Pepper ftonecrop, botanical defcription of 265
Pericardii, analyfis of the liquor 281
Perkinifm, on the quackery of 369
Peffary, on the ufe of the fponge 21, 235
Phlegmafia alba, on the 5
Phthifis cured by uva urfi 346
Pinckard's, Dr. account of the expedition to the Weft Indies 296
Plague at Conftantinople, account of 113
Plants, inftance of the fleep of 264
———, botanical defcription of Britifh 66, 261
Pliny, character of 379
Portland powder, on the 194
Poifons, on morbid 275
Poucqueville, M. on the plague 113
Powell's, Dr. lectures announced 94
Procidentia uteri, cafe of incipient 21
Proftate gland, particular affection of 487
Pfora, cafe of 579
Pully's, M. analyfis of James's powder 294
Pulvis bafilicus, on the 432
Purgative medicines, on 182
Puftule, on fpurious 308

Quacks, anecdotes of 366, 370
Quackery, obfervations on 334
Quebec, ftate of vaccination at 313
———, on a malignant fever at 446

Ragufa, fuccefs of vaccination at 390
Rainbird, Mr. on the febris intermittens 333
Reform, on medical 258, 556
Reid's, Dr. report of difeafes 90, 193, 291
———, lectures announced 96, 487
Reid's, Dr. treatife on confumption announced 96; reviewed 160
Remittent fever, account of a 189
Rheumatifm, cafe of acute 45
———, difference between gout and 177
———, on the cure of 484
Richter's, Dr. account of a new metal 390
Ring, Mr. on inoculation 56
——— on Rowley's Pamphlet 62, 157
——— his anfwer to Mofeley 191
——— on vaccination 167, 310, 516
——— cafes of fecondary fmall-pox 482
Rofewort, botanical defcripti n of 70
Roberton, Mr on cantharides in gleet and leucorrhœa 415
Rock ftonecrop, botanical description of 266
Rook major, cafe of 181
Roots, on the defcent of 295
Rotheram, Dr. on the palfy 47
Rouffeau's, Mr. experiments on cutaneous abforption 274
Rowley, Dr. anfwers to 62, 157, 231, 286, 290, 445, 527
Royfton's, Mr. cafes of cafual fmall-pox 443
——— on medical literature announced 583
Rufh's, Dr. works announced 96
——— recantation on yellow fever 271
Rufpini's, Mr. Inftrument for extracting balls 455

Sacco, Dr. on vaccination 524
Saliva, analyfis of 281
Sanders's Mr. prefent from the ftudents to 584
——— anatomy of the ear, announced 199
Scabies, remedy for 270
Scalds, on the treatment of 342
———, on cold water in 458
Scarlatina anginofa, on the 489
———, utility of purgatives in 185
Schuppach, a quack, account of 364
Scirrhi, on the treatment of 80
Scott, Mr. on vaccination 245
Scrofula, on 76
Scrotal hernia, cafe of 381
Scurvy, remedy for the 70
———, benefit of wood-forrel in 267
Shearly, Mr. on gun-fhot wounds 97
Sheep, inoculation of 517
———, on the difeafes of 518
Sibbens, a difeafe in Scotland 26
Sims's Dr. theory of cow-pock 571
Simmons, Mr. on felect fubjects in furgery 1
Simpfon's, Mr. cafe of ulcerated leg 21
——— cafes of fecondary fmall-pox 219

Sims, Dr. on the fpurious cow-pox 168
Sleep of plants, on the 264
Small-pox and inoculation hofpitals, report of the 195
———— cafes of 233
———— inoculation 305
———— cafes of fecondary 433
———— cafes of confluent 532
Smith, Dr. on the report of M. Chaptal 288
———— reply to 373
———— on lithotomy 569
——, Mr. his cafes of croup, &c. 382
Snakeweed, defcription and ufes of 66
Snuff, bafis of cephalic 268
Somerfet Jennerian Society, report of the 309
Sores, remedy for ulcerous 265
Spafms of the ftomach, remedy for 563
Speech, on difficulty of 172
Sphacelus, on the ufe of nitre in 504
Spina bifida, cafes of 236
Spleen, remedy for the 270
Sponge peffary, advantages of the 21, 235
Spry, Mr. on a peculiar morbid appearance in the heart 386
Sputa of confumptive perfons, on 20
Stearoid tumour, analyfis of a 276
Sternutatory, account of a 268
Stewart's, Dr. cafes of inoculation 58
Stock, Dr. on cutaneous abforption 274
Stomach, on the difeafes of the 576
Stomach, remedy for fpafms of 563
Stone Dr. on the difeafes of the ftomach 576
Stone and gravel, cafe of 35
Struve's, Dr. galvanic machine, account of 295
Sulphur ftibiatum aurant, ufe of 32
Surgery, on felect fubjects in 1
Sutton's, Dr. account of a remittent fever 189
————, Mr. on vaccination 166
Sydenham on chorea 124
Syphilis, on the diagnofis of 22
————, cured by bruifewort 263

Tanning, on artificial 295
Tape worm, on arfenic in cafes of 300
Tartar of the teeth, analyfis of the 372
Taunton's, Mr. lectures announced 96
Taynton, Mr. on vaccination 245
Teeth, review of Mr. Fox's treatife on the 371
Terebinth. ol. its ufe in burns 146
Tetanus, cafe of 496
Thelwall, Mr. on difficulty of fpeech 172
———— his lectures announced 392
Thigh, on fractures of the 8, 339
Thomas, Mr. on Dr. Cuming's paper 36
————, reply to 224
———— W. on the treatment of fcirrhi and cancers 79
Thomas, Dr. in anfwer to Mr. Fogo 141
———— cafe of a blue boy 381
————'s, St. hofpital, lectures at 198
Thornton's, Dr. Vaccinae Vindicia, reviewed 290
———— on oxygen gas 582
Thynne's, Dr lectures announced 95
Throat, cafes of fore 485
Tinea capitis, cafe of 43
Tooth-ache, remedy for the 262
Torpedo, experiments on the 295
Tourniquet, on the fcrew 7, 112, 149
Trifmus, cafe of 43
Trotter's, Dr propofal for deftroying damps in coal mines 96
———— work on indigeftions account of 213
————, on gaftrodynia 216
Trye, Mr. on cow-pox and fmall-pox 301
Tubercles, on fœtid 30
Tumour, analyfis of a ftearoid 276
Turpentine, on the fpirit of, in yellow fever 16
————, experiments on 275
Tuffis convulfiva, cafes of 222
Typhus fever, on purgatives in 183
————, cafes of 239, 292

Ulcerated leg, cafe of 217

Ulceration following venæfection, cafe of 348

————, cafe of inteftinal 386

Ulcers, fuccefsful application to fcrophulous 267

Urine cafter, droll anecdote of a 365

Uterine fœtus, cafe of extra 385

Uterus, cafe of offified 279

————, cafe of inverted 385

Uva urfi, its effects in phthifis 346

Valentin, Dr. on vaccination 516
Vaccination, Dr. Walker on 544
————, on the irregular adminiftration of 580
Vaccination, addrefs on 92
————, defence of 157, 191, 231, 245, 283, 290, 301
————, fuccefs of 195
————, Dr. Clarke on 254, 426
————, Mr. Creafer on 306
———— in America, account of 311
———— in France, ftate of 314
————, Mr. Ring on 516
Vaccine inoculation, report on 107
————, Dr. Wood on 135
————, cafes of 234, 246
————, at Nottingham, inftitution to promote 251, 426
————, obfervations on 350
———— at Ragufa, ftate of 390
————, obfervations on 350
———— at Liverpool, inftitution at 536
Vaccine veficle, on rupturing the 238

Variolous and vaccine difeafe, on the identity of 48
——————, on the radical diverfity of the 219
Varnifh, account of a new 391
Venæfection, cafe of ulceration following 348
Venereal complaints, ufe of bruifewort in 263
——————, effects of arfenical folution in 299
Verral's, Mr. cafe of fecondary fmall-pox 436
Vifcera, on the difeafes of the abdominal 481, 573
Vifceral obftructions, remedy for 270

Wachfell's, Mr. account of patients in the fmall-pox hofpitals 198
Waiblinger, Mr. on the effects of galvanifm 150
Waldon, Dr. on vaccination 526
Wales, (New South) vaccination in 523
Walker's Dr. reply to Mr. Ring 544
——— anfwer to Dr Clarke 546
Wallis, Dr. on impediments of fpeech 172
Walfh, Dr. on a malignant fever at Quebec 446
Water, obfervations on 75
———, on cold, in convulfions 52
——————, in burns and fcalds 458
Weaver's, Mr. cafe of apoplexia hydrocephalica 322
—————— cafe of periodic head-ach 506
Weft Indies, on the climate and difeafes of 41
Whitlam, Mr. on the agaricus campeftris 247
Whittle, Mr. on the couching needle 103
Whitworth doctors, practices of 553
Willan, Dr. on the cow-pox announced 488
———, letter from Dr. Binns to 400
Willan's Dr. work on cow-pock, account of 584
Wine, on the ufe of in fcarlatina anginofa 494
Witte's, Dr. cafe of hydrocephalus internus 28
Wood, Dr. on vaccine inoculation 135
———, his cafes of confluent fmall-pox 532
Wood-forrel, conferve made from 267
———, defcription and virtues of 266
Worm fhell, account of a 391
Worms, on imputing children's complaints to 185
Wounds, on the ufe of nitre in gun-fhot 95
———, inftrument for extracting balls from 455
Woollett, Mr. on the difeafes of fheep 518

Yarmouth, addrefs to the inhabitants of 92
Yellolv's, Dr. lectures announced 94
Yellow fever, on fpirit of turpentine in 16
———, effects of cold bathing in 17
———, Dr. Rufh's recantation on 270
———, on the contagious nature of 560
Young, Mr. on cancer 393
——— his letter to Mr. Home 500

Zinc in chorea fancti, on 127
———, its effect on metallic oxyds 295

### REFERENCES TO THE PLATES.

The Ball which killed Lord Nelson – – – – – – – – 72
Mr. Shearly's Case of Gun-Shot Wound – – – – – 97
Dr. Gall's Craniology – – – – – – – – – – – – – 201
Mr. Ruspini's Instruments for the Extraction of Balls – 455

